# Influence of Microencapsulated Phase Change Material (PCM) Addition on (Micro) Mechanical Properties of Cement Paste

**DOI:** 10.3390/ma10080863

**Published:** 2017-07-27

**Authors:** Branko Šavija, Hongzhi Zhang, Erik Schlangen

**Affiliations:** Microlab, Delft University of Technology, 2628 CN Delft, The Netherlands; b.savija@tudelft.nl (B.Š.); erik.schlangen@tudelft.nl (E.S.)

**Keywords:** cement paste, nanoindentation, PCM, microcapsules, tensile strength, porosity

## Abstract

Excessive cracking can be a serious durability problem for reinforced concrete structures. In recent years, addition of microencapsulated phase change materials (PCMs) to concrete has been proposed as a possible solution to crack formation related to temperature gradients. However, the addition of PCM microcapsules to cementitious materials can have some drawbacks, mainly related to strength reduction. In this work, a range of experimental techniques has been used to characterize the microcapsules and their effect on properties of composite cement pastes. On the capsule level, it was shown that they are spherical, enabling good distribution in the material during the mixing process. Force needed to break the microcapsules was shown to depend on the capsule diameter and the temperature, i.e., whether it is below or above the phase change temperature. On the cement paste level, a marked drop of compressive strength with increasing PCM inclusion level was observed. The indentation modulus has also shown to decrease, probably due to the capsules themselves, and to a lesser extent due to changes in porosity caused by their inclusion. Finally, a novel micro-cube splitting technique was used to characterize the tensile strength of the material on the micro-meter length scale. It was shown that the strength decreases with increasing PCM inclusion percentage, but this is accompanied by a decrease in measurement variability. This study will contribute to future developments of cementitious composites incorporating phase change materials for a variety of applications.

## 1. Introduction

Reinforced concrete is a construction material of choice for structures built in challenging environments. Compared to materials such as steel and timber, it has relatively good properties in aggressive conditions, leading to its high durability. Nevertheless, concrete is a quasi-brittle material susceptible to cracking due to mechanical and environmental loading [1]. Cracks in the concrete can promote deterioration by allowing rapid ingress of chloride [2] or carbon dioxide [3]. This will then lead to a fast corrosion initiation and propagation [4]. It is therefore of practical importance to avoid the occurrence of excessive cracking.

There are different strategies of achieving this, depending on the underlying cause of cracking. For example, if the mechanical loading is the cause, cracking can be limited by using fiber reinforcement, for example polyvinyl alcohol (PVA) fibers [5,6]. On the other hand, if the cracking is caused by thermal variations (such as early age temperature rise due to cement hydration or freeze-thaw damage), controlling the temperature is a good option. To achieve this, incorporation of phase change materials (PCMs) has been proposed in the past few years [7,8,9,10]. Phase change materials are combined (sensible-and-latent) thermal storage materials that can store and dissipate energy in the form of heat [8]. PCMs are usually added to the concrete mix as either microencapsulated particles [8], within embedded pipes [11], or as part of lightweight aggregates [12].

When microencapsulated PCMs are added to the mix, they influence the mechanical properties of the cement paste and, consequently, concrete [8,9,13,14]. This is (presumably) because the microcapsules are softer than the matrix material. The decrease of compressive strength seems to be more pronounced than of tensile strength [8,9,13].

In this study, the effect of PCM microcapsule addition on micromechanical properties of cement pastes is studied. Micromechanical testing of both the capsules and the composite paste is performed together with various characterization techniques. The focus is on a newly developed experimental technique based on nanoindentation, namely microcube testing [15]. This technique enables mechanical testing of cement paste on the representative length scale, i.e., the micrometer scale. This study provides insight into causes of changes in mechanical properties and their practical implications.

## 2. Experimental Program

### 2.1. Materials

In order to quantify the influence of microcapsule addition on the mechanical properties of cement based materials, studies were performed on the binding phase of concrete, i.e., the cement paste. For the purpose of material characterization, cement paste specimens were prepared. All pastes used ordinary Portland cement (CEM I 42.5 N) as a binder and a water-to-cement ratio of 0.45. Four different mixtures were used, with different levels of PCM addition: a reference mixture and mixtures containing 10%, 20%, and 30% of PCM microcapsules per volume, respectively. 

Microcapsules used in this study are composed of a paraffinic phase change core and a melamine formaldehyde (MF) shell, with a core-to-shell ratio (mass based) of around 11.8 [13]. Enthalpy of phase change provided by the manufacturer was 143.5 J/g and the median particle size 22.53 µm.

The pastes were mixed in accordance with EN 196-3:2005+A1:2008 (E) using a Hobart mixer. First, the dry material (cement and PCM powder) was placed in a bowl. Water was added within 10 s. This was followed by mixing for 90 s at low speed. The mixer was then stopped for 30 s during which all paste adhering to the wall and the bottom part of the bowl was scrapped using a metal scraper and added to the mix. The mixing was then resumed for additional 90 s. The total mixer running time was around 3 min. The mix was then cast in plastic cylinders with an inner diameter of 34 mm and height of 58 mm. The cylinders were then sealed and rotated slowly for around 24 h in order to avoid bleeding. The pastes were then cured in sealed conditions until needed.

### 2.2. Microcapsule Characterization

#### 2.2.1. Differential Scanning Calorimetry (DSC)

Differential scanning calorimetry (DSC) was used to investigate the thermal properties of microencapsulated PCMs. This included the onset and peak temperatures and enthalpy. The thermal program was as follows: the sample was heated from −20 °C to 100 °C and then cooled back to −20 °C in a nitrogen environment. The rate of heating and cooling was set to 5 °C per min.

#### 2.2.2. Scanning Electron Microscopy (SEM)

The microstructure of microencapsulated PCMs was observed using a Philips XL30 Environmental Scanning Electron Microscope (ESEM) (FEI, Eindhoven, The Netherlands). The microcapsules were sprinkled on top of a glass plate which was coated with superglue to ensure bonding, and were subsequently imaged in the secondary electron (SE) mode.

#### 2.2.3. Particle Size Distribution

The particle size distribution and the mean particle size of PCM microcapsules were determined by laser diffraction.

#### 2.2.4. Compression of Microcapsules

**Figure 1:**
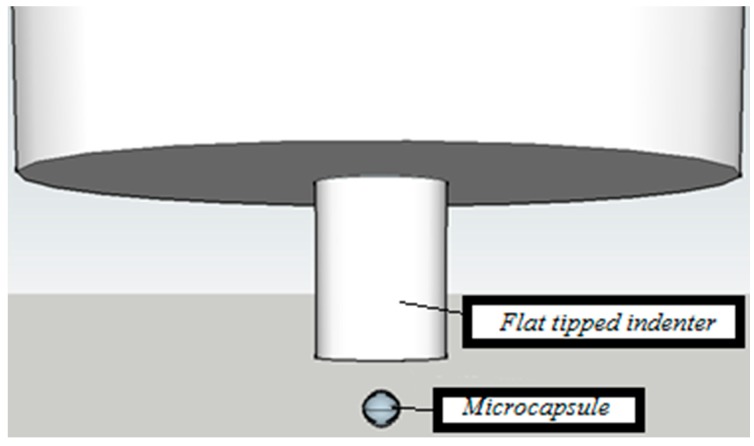
Nanoindentation setup used to measure the force-displacement relationship of phase change material (PCM) microcapsules.

In order to examine the influence of temperature on the mechanical properties of microcapsules, compression of individual microcapsules was performed. Microcapsules were sprinkled on top of a stage and individual microcapsules were identified and subjected to loading using a flat tiped indenter with a diameter of 135 µm (Figure 1). This method was initially proposed by the authors of [16]. This was done for room-temperature conditions (>25 °C, above the phase change temperature as determined by DSC), and for 15 °C using a temperature stage (below the phase change temperature). The relationship between maximum load and capsule diameter was determined for both temperatures.

### 2.3. Cement Paste Characterization

#### 2.3.1. Compressive Strength Development

Compressive strength of cement paste specimens with different levels of PCM addition was measured at 1 day, 3 days, 7 days, 14 days, and 28 days. Cylindrical paste specimens with a diameter of 34 mm were cut to height of 40 mm (by cutting off the ends of the 58 mm high cylinder) and exposed to uniaxial compression. The loading rate of 1 kN/s was applied until specimen failure. Three specimens were tested for each condition.

#### 2.3.2. Porosity and Pore Size Distribution

Porosity of the cement paste samples was determined using Mercury Intrusion Porosimetry (MIP). MIP is a commonly used technique for porosity investigation of cement based materials [17]. Although heavily criticized [18], this technique can be considered appropriate for comparative purposes as used herein.

For testing, a Micrometrics PoroSizer 9320 (Micrometrics, Norcross, GA, USA) device was used with a maximum pressure of 207 MPa. The contact angle and the surface tension of the mercury were set to 139°and 485 mN/m, respectively.

Prior to testing, hydration of the specimens was stopped using solvent exchange by isopropanol [19]. Paste specimens (obtained from cylinders by cutting) were submerged five times and taken out for a period of one min in order to enable a fast exchange of water and the solvent. Afterwards, they were placed in isopropanol for a prolonged period of time. This was followed by crushing and then vacuum drying for at least three months until the specimens were completely dry (determined by monitoring their weight over time).

Porosity and pore size distribution was measured at 3, 7, and 28 days for each cement paste mix.

#### 2.3.3. Nanoindentation Testing

Micromechanical properties of cement paste mixtures with different additions of PCM microcapsules were measured by nanoindentation technique. Nanoindentation enables determination of local mechanical properties of tested volumes from the indentation load/displacement curve [20]. In the past decade, it has been commonly used to investigate the micromechanical properties of cementitious materials [21,22,23,24,25].

The elastic modulus of the indented material can be obtained from the following Equation:

(1) $$\frac{1}{E_{r}}=\frac{1-\nu_{s}^{2}}{E_{s}}+\frac{1-\nu_{i}^{2}}{E_{i}}$$

where *ν_s_* is the Poisson’s ratio of the tested material, *ν_i_* the Poisson’s ratio of the indenter (0.07), *E_s_* the Young’s modulus of the sample and *E_i_* the Young’s modulus of the indenter (1141 GPa). It is assumed that during the unloading phase only elastic displacements are recovered, and that the reduced elastic modulus, *E_r_*, can be determined using the slope of the unloading curve:

(2) $$S=\frac{dP}{dh}=\frac{2}{\sqrt{\pi }}E_{r}\sqrt{A}$$

Here, *S* is the elastic unloading stiffness defined as the slope of the upper portion of the unloading curve during the initial stages of unloading, *P* is the load, *h* the displacement relative to the initial undeformed surface, and *A* the projected contact area at the peak load.

Nanoindentation tests were performed for specimens after 28 days of hydration. Prior to testing, hydration was stopped as described. Discs cut from the cylindrical pastes were glued onto a glass holder. The specimens were ground using sandpaper, during which ethanol was used as a cooling liquid. After grinding, samples were polished with 6 µm (5 min), 3 µm (5 min), 1 µm (10 min), and 0.25 µm (30 min) diamond paste on a lapping table. After each polishing step, samples were soaked into an ultrasonic bath to remove any residue. Sample preparation was performed just prior to testing to avoid carbonation of the tested surface.

An Agilent Nanoindenter G200 (Keysight, Santa Rosa, CA, USA) equipped with a diamond Berkovich tip was used for nanoindentation. For each specimen, a series of 20 × 20 indents were performed on a tightly spaced grid, with spacing of 20 µm between indents. Indentation depth was set to 700 nm. The Continuous Stiffness Method (CSM) proposed by Oliver and Pharr [20], which provides continuous measurements of elastic modulus as a function of indentation depth, was used to analyze the results. The average E modulus was determined in the loading range between 500–650 nm. For the calculation, Poisson’s ratio of the indented material was taken as 0.18.

#### 2.3.4. Microcube Splitting

While nanoindentation can be considered appropriate for measuring the elastic properties of cement paste and its individual phases, more complex procedures are needed for measuring strength properties at the micrometer length scale. This is because no relation between the indentation hardness and strength has been found so far for cement based materials [15,26]. Therefore, more advanced procedures that use e.g., nanoindentation equipment need to be used.

Recently, several authors have proposed measuring the tensile strength of cement paste [27] and it’s individual phases [28] using micro-cantilever bending tests. This technique has been previously used for micromechanical testing of other quasi-brittle materials such as e.g., nuclear graphite [29,30]. This technique involves focused ion milling of a cantilever beam in the material, typically in the size range of up to 10 µm. Such cantilever beam is subsequently loaded in bending and tested until failure, providing a measure of the elastic modulus and the flexural strength of the tested microvolume. A major drawback of this approach is the fact that specimen preparation is very time consuming, so a relatively small number of specimens can be prepared and analyzed. Keeping in mind that on the µm length scale high scatter of measured mechanical properties can be expected [29,30] and that a large number of tests need to be performed for the measurements to be statistically reliable, herein a different approach is followed.

**Figure 2:**
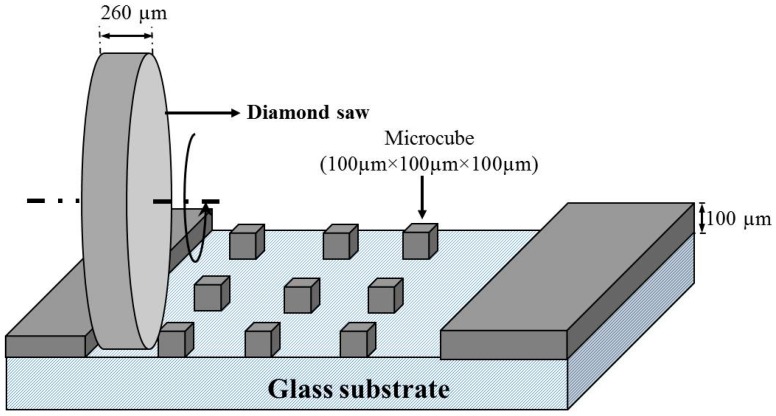
A schematic view of the specimen preparation procedure [15].

**Figure 3:**
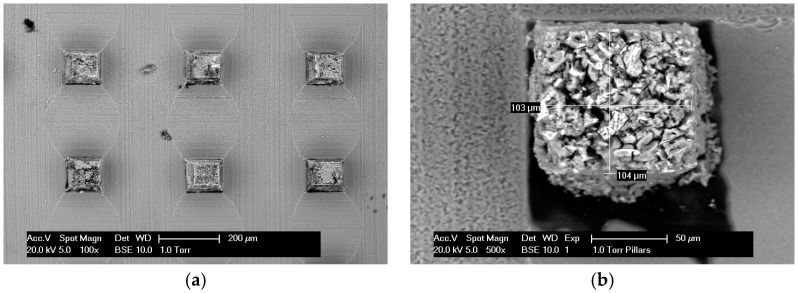
SEM images (**a**) A grid of micro-cubes on a glass substrate; (**b**) A single microcube.

In this work, a recently developed method for creating a grid of micro-cubes (100 × 100 × 100 µm), developed by the authors of [15], is used. The method is shortly presented here. Cement paste specimens aged 28 days (with the hydration halted as previously described) were first glued on top of a glass substrate. Then, it is necessary to make the specimen thickness equal to the desired thickness (100 µm in this case), and this was done using a Struers Labopol-5 thin sectioning machine. The specimen is then ready for creation of the micro-cube grid. This is done using a precise diamond saw (MicroAce Series 3, Loadpoint, Swindon, UK) that is commonly employed in the semiconductor industry to create silicon wafers. To prevent chipping of the edges of the micro-cubes during cutting, a thin layer of soluble glue was applied on the surface of the thin section, which was later removed by soaking the specimen for a short time in acetone. In the machine, a 260 µm thick blade was run in two perpendicular directions over the specimen and the glass substrate (Figure 2). The procedure results in a grid of micro-cubes (100 × 100 × 100 ± 4 µm) that are used for micromechanical testing (Figure 3).

**Figure 4:**
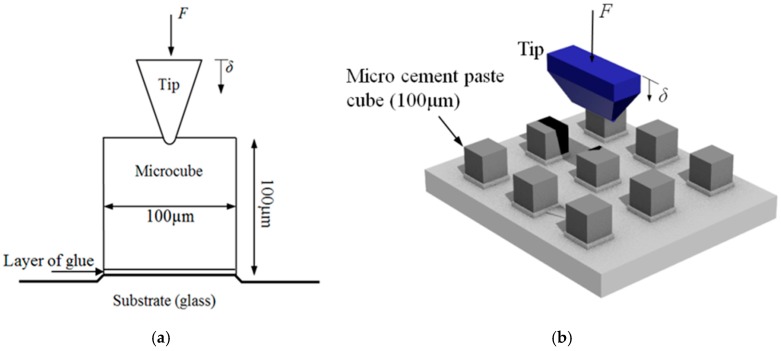
Schematic illustration of (**a**) A contact between the indenter tip and a single microcube; (**b**) the knife-tip loading procedure.

For testing of the micro-cubes, the nanoindenter is employed (Figure 4). For the purpose of this splitting test, a diamond cylindrical wedge tip (radius 9.6 µm, length 200 µm) was used in order to apply the load across the middle axis. The experiments were run using displacement control with a loading rate of 50 nm/s.

## 3. Results and Discussion

### 3.1. Microcapsule Characterization Results

**Figure 5:**
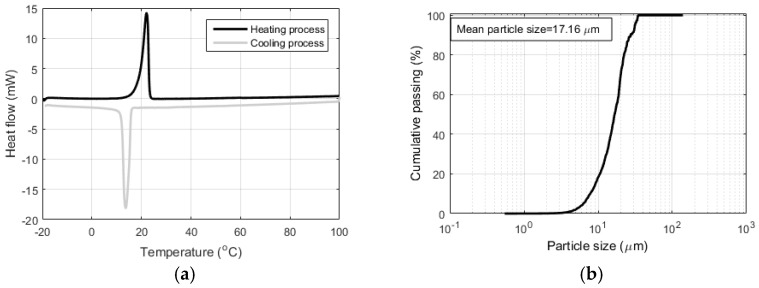
(**a**) Differential scanning calorimetry (DSC) thermograph of the PCM microcapsules; (**b**) Particle size distribution of microencapsulated PCM. (Adapted from study [9]).

Differential scanning calorimetry (DSC) curves of the PCM microcapsules are shown in Figure 5a for both the heating and the cooling regime. The heat of fusion during the phase change was determined as the area under the heat flow curve during the phase transition. Measured heat of fusion was 146.7 J/g, which corresponds well with the value provided by the manufacturer (143.5 J/g). The onset of phase change corresponding to melting is measured at 19.07 °C, and the endothermic peak at 22.07 °C. Considering their phase change temperature, these particles are suitable for applications such as reduction of temperature rise in young concrete for structures cast in moderate climatic conditions, as shown by previous finite element (FE) analyses by the authors [10] and others [31,32].

**Figure 6:**
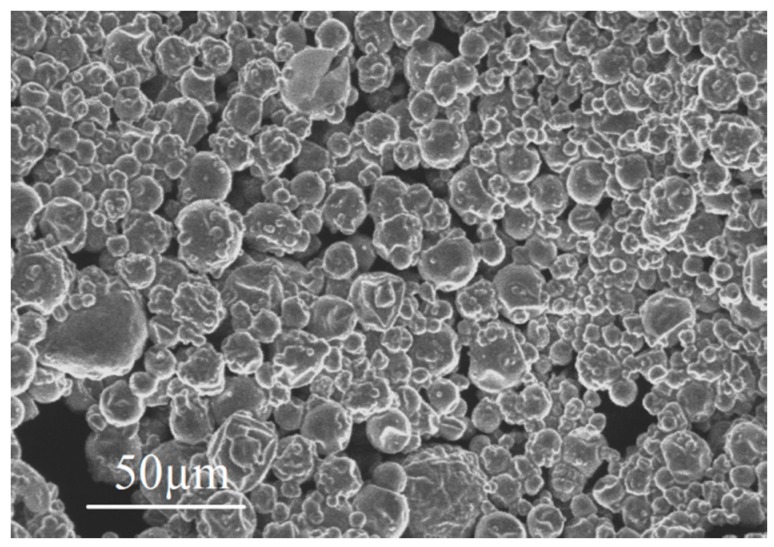
A micrograph of dispersed PCM microcapsules (adapted from [9]).

In order to observe individual PCM microcapsules, they were sprinkled on a superglue layer on top of a glass substrate and placed inside the ESEM chamber. Imaging was performed using the secondary electron mode, acceleration voltage of 7 kV and 200× magnification. A micrograph of microencapsulated PCMs is shown in Figure 6. It can be seen that microcapsules are spherical in shape with a range of different diameters, which is beneficial for proper dispersion inside the cementitious matrix, as shown by [13] using micro-computed X-ray tomography.

Particle size distribution of the microcapsules is shown in Figure 5b, with a mean particle size of 17.16 µm, which is somewhat smaller than reported by the manufacturer (22.53 µm).

**Figure 7:**
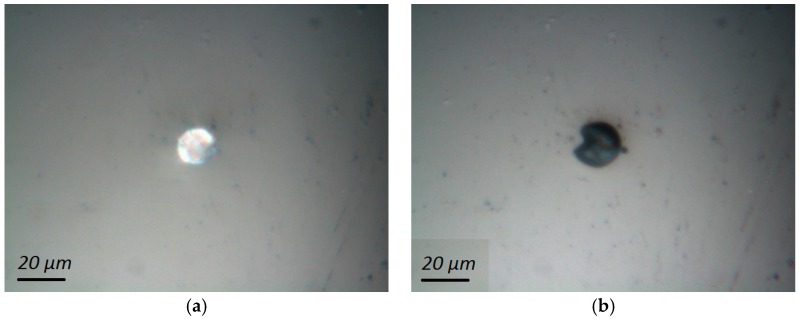
An individual microcapsule imaged in the nanoindenter (**a**) before compression testing; (**b**) after compression testing.

Compression testing of individual microcapsules was performed as described previously. An example of a microcapsule before and after compression testing is shown in Figure 7. As the capsule ruptures, the encapsulated content (paraffin wax that acts as a phase change material in this case) is squeezed out, as seen in Figure 7b.

**Figure 8:**
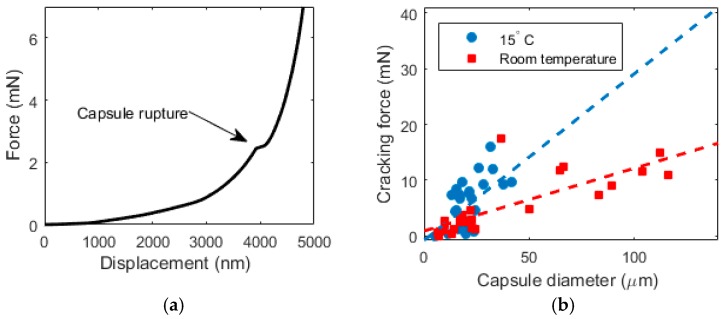
(**a**) A typical load vs. displacement curve measured in the capsule compression test; (**b**) A relationship between capsule diameter and cracking force for capsules below and above the phase change temperature (dashed lines indicate a linear fit).

While it is possible to relate the rupture force with the capsule diameter when punching with a Berkovich indenter is used, as shown by [33], herein the approach using a flat indenter tip was used. The method was initially described by [16] who tested brittle microcapsules (microballons) that show a distinct plateau in the load-displacement curve, indicating capsule failure. Since the capsules tested herein are not brittle, the identification of “failure” load was not as simple. A typical force-displacement curve of a microcapsule is shown in Figure 8a. However, the bump in the curve was not always visible, so it was not possible to determine the rupture force for all tested microcapsules. In the analysis provided, only capsules with a clear rupture point are included. Diameters of individual microcapsules are measured using microscopic images taken in the nanoindenter before the testing, such as the one shown in Figure 7a.

It is clear that the rupture force of the microcapsules exhibits a size dependence, as capsules with larger diameters clearly require more force to rupture Figure 8b. It is also interesting that the capsule strength exhibit temperature dependence, as the capsules tested below the phase change temperature (at 15 °C) need a higher rupture force compared to those tested at room temperature (above 25 °C). This is because the encapsulated material seems to contribute to the load bearing capacity when it is in the solid phase, but not when it is in the liquid phase. Although the influence of temperature on the mechanical properties of the cement paste with microencapsulated PCM addition was not tested here, it will be a part of further research.

### 3.2. Cement Paste Characterization Results

#### 3.2.1. Compressive Strength Results

**Figure 9:**
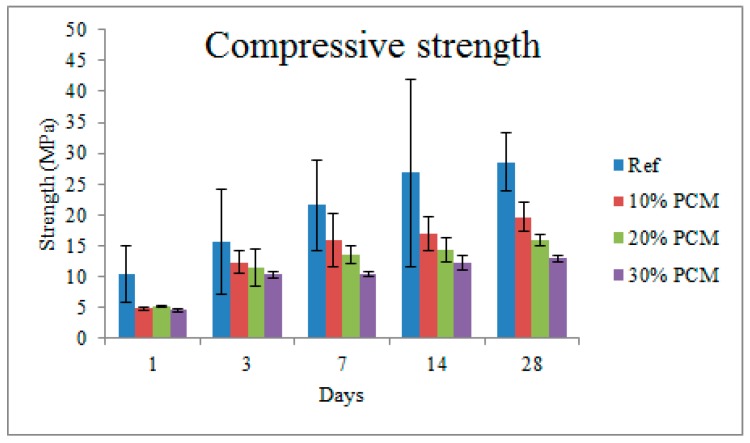
Development of paste compressive strength as a function of PCM addition percentage (error bars indicate standard deviation).

The development of compressive strengths as a function of time for cement pastes with different percentages of microencapsulated PCM additions is given in Figure 9.

After 1 day, there is a marked difference between the strength of plain cement paste and the pastes with incorporation of PCM microcapsules. However, at this age, there is no significant difference between specimens with different amounts of PCM microcapsules. This changes already after 3 days, when a clear decrease of compressive strength with PCM addition percentage is observed. This trend remains valid until 28 days, when the compressive strength decreases by 31.2%, 44.5%, and 54.8% for the 10%, 20%, and 30% volumetric PCM inclusion, respectively.

#### 3.2.2. Porosity Measurements

**Figure 10:**
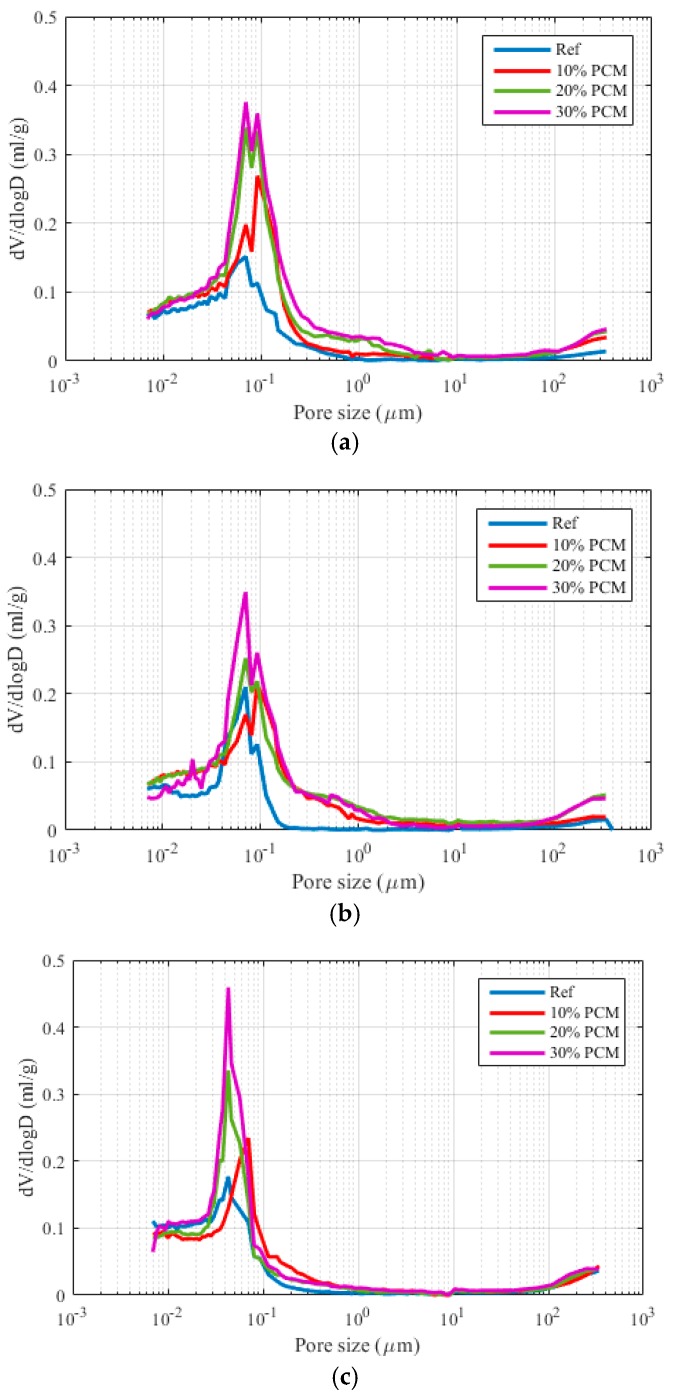
The effect of PCM microcapsule addition on the pore size distribution in cement paste samples after (**a**) 3 days; (**b**) 7 days and (**c**) 28 days of hydration.

**Figure 11:**
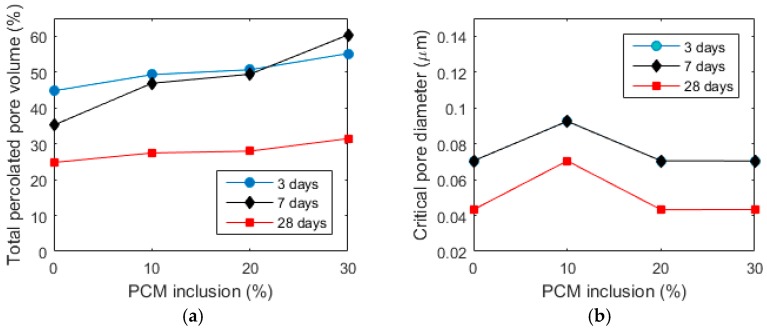
(**a**) Total percolated pore volume and (**b**) Critical pore diameter for cement pastes with various PCM inclusion percentages at different ages.

In Figure 10, pore size distributions for pastes aged 3, 7, and 28 days with varying PCM inclusion percentages are given. From these curves, critical pore diameters are extracted as follows: a peak in the differential PSD curve is defined as the critical pore diameter [17,34]. If two peaks are observed (such is the case with 30% PCM sample after 3 days of hydration), then the highest peak is defined as the critical pore diameter. MIP measurements provide, in addition, a measure of the total percolated pore volume. The percolated pore volume and critical pore diameter for all the pastes are given in Figure 11.

It can be observed that the total percolated pore volume increases with the increase in PCM dosage, more so for early hydration ages (3 and 7 days), and significantly less for the age of 28 days. Therefore, it is unlikely that the strength of the composite pastes is influenced by the increase in porosity caused by microencapsulated PCM addition. It is probable that the major cause of the strength drop is the addition of weak inclusions in the form of PCM microcapsules.

The critical pore diameter, on the other hand, remains constant for different paste formulations at the same age (apart from the 10% specimen, which shows a somewhat larger critical pore diameter). For the 3 and 7 day old pastes, the critical pore diameters do not change for any of the paste formulations. The critical pore diameters do decrease for the 28 day formulations. It needs to be noted that the critical pore diameter is a controlling parameter for durability of concrete. For example, chloride diffusion coefficient shows a linear relationship with the critical pore diameter, while the permeability shows a power relationship [35]. Furthermore, a recent study has shown that the addition of microencapsulated PCMs has very little influence on water absorption [36]. It is important that the long term durability of the cement paste with incorporated microencapsulated PCMs will not be affected.

#### 3.2.3. Nanoindentation Results

**Figure 12:**
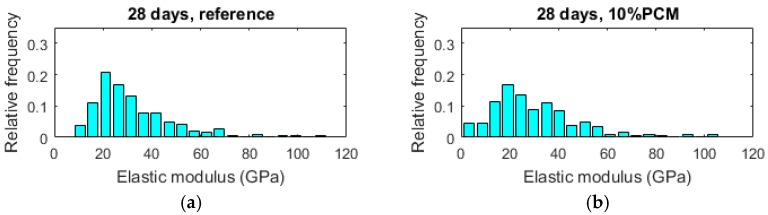

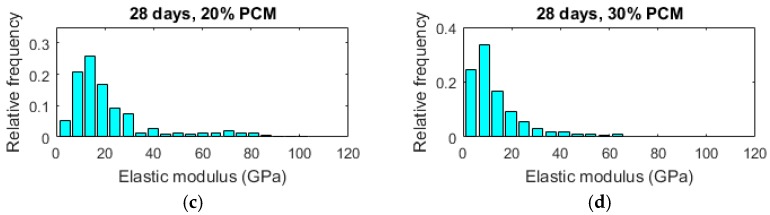
Histograms of elastic moduli for pastes with various percentage of PCM microcapsules measured by nanoindentation. (**a**) 28 days, reference paste; (**b**) 28 days, 10% PCM paste; (**c**) 28 days, 20% PCM paste; (**d**) 28 days, 30% paste.

In Figure 12, histograms of measured elastic modulus for 28-day old pastes with various percentages of PCM inclusions are given. As the percentage of PCM microcapsules increases it can be seen that histograms shift towards lower elastic modulus values. Although PCM microcapsules at the specimen surface are likely to be damaged during the specimen preparation procedure, the moduli reduced with PCM inclusion percentage increase. This is because nanoindentation is a volumetric measurement: a test will sample the material under the indenter up to a certain depth depending on different factors, as shown by [26]. Since MIP measurement showed no significant increase in porosity of the paste phase, this is most probably the reason.

**Figure 13:**
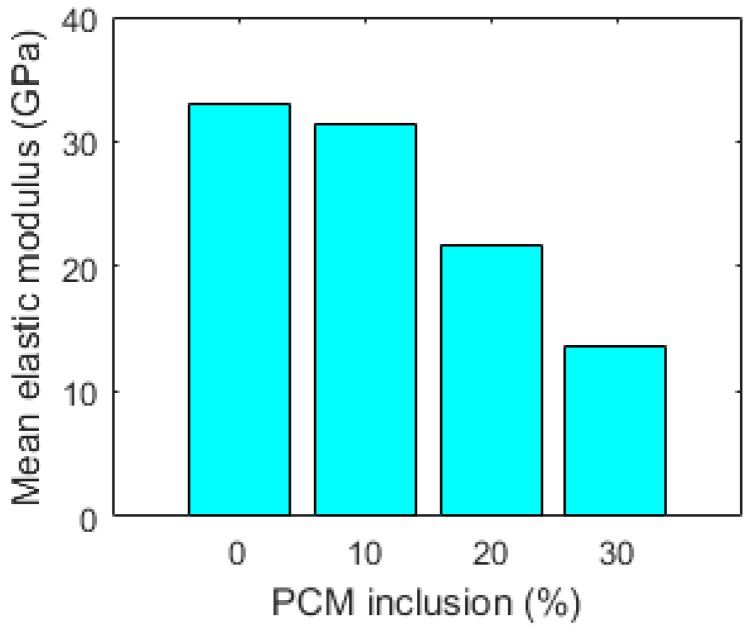
Mean elastic modulus of cement paste as a function of PCM volume fraction, measured by nanoindentation.

In Figure 13, mean values of elastic modulus for pastes with increasing percentages of PCM inclusions are given. It is clear that the mean elastic modulus decreases with the increased amount of PCM inclusions in the mix. This is expected, as the addition of relatively large inclusions was previously shown to linearly decrease the elastic modulus with increasing inclusion volume in model quasi-brittle materials [37]. The mean elastic modulus measured by nanoindentation decreases by 6.1%, 33.9%, and 58.8% for the 10%, 20%, and 30% volumetric PCM inclusion, respectively, compared to the reference. While the decrease in strength may be considered detrimental for structural use of cementitious materials, a decrease of the elastic modulus may be beneficial for certain applications of crack control, since it will lead to lower stress build up in e.g., restrained deformation condition [8,10].

#### 3.2.4. Microcube Splitting Results

**Figure 14:**
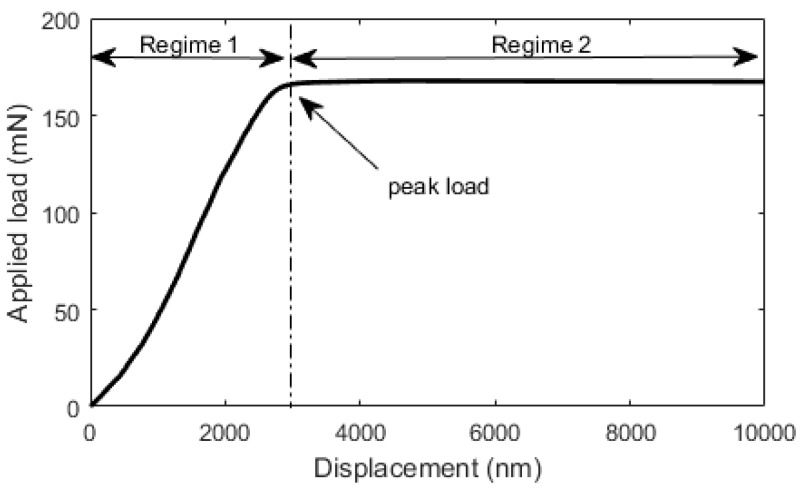
A typical load vs. displacement curve measured in the microcube splitting test.

The micro-cube splitting test performed in this work results in a load vs. displacement curve for a tested micro-cube. A typical load-displacement curve is shown in Figure 14. The curve shows two distinct regimes. Regime 1 signifies a nearly linear load-displacement curve until the peak is reached. After the peak load, the system enters an unstable regime (regime 2), which signifies a rapid crack propagation and failure of the micro-cube. Due to limitations in speed of the displacement control, the post-peak behaviour cannot be measured at present.

**Figure 15:**
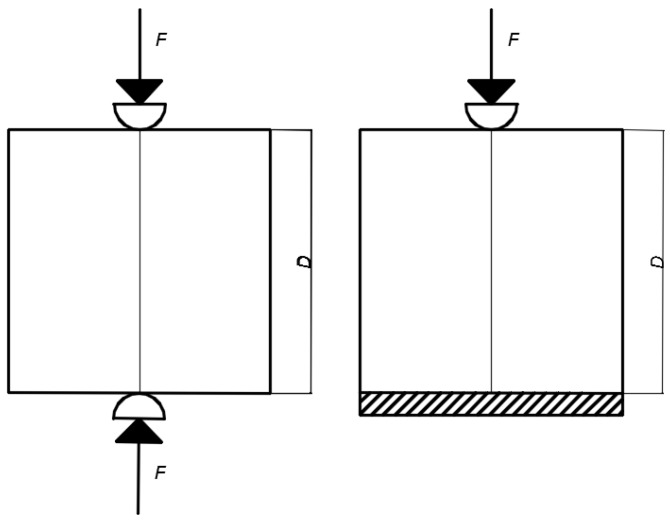
Schematics of the Brazilian splitting test (**left**) and the microcube splitting test (**right**). *D* is the specimen height, and *F* is the applied force.

The setup of the micro-cube splitting test is similar to the Brazilian test (NEN-EN 12390-6 Standard) for splitting tensile strength assessment of cement based materials. The difference is in the boundary condition at the bottom: in the standard Brazilian test, a linear support is used. In the micro-cube splitting test, the specimen is clamped (glued) to the bottom (Figure 15).

In the Brazilian splitting test, a line load is applied on the top and the bottom surface of the specimen, leading to an almost uniform distribution of horizontal splitting stresses in the middle of the specimen. The magnitude of failure splitting stress can be determined using linear elastic theory as [38]:

(3) $$f_{st}=\frac{2P}{\pi D^{2}}$$

In Equation (3), *P* is the maximum load, and *D* the specimen height. In the micro-cube splitting test, the bottom side is glued to the glass plate, leading to a somewhat different stress distribution. As shown by Zhang et al. [39], a modification of Equation (3) can be used to calculate the splitting stress in this case as:

(4) $$f_{st}=0.73\cdot \frac{2P}{\pi D^{2}}$$

For the tests performed herein, Equation (4) was used to calculate the splitting strength of micro-cubes based on the peak load *P*, as shown in Figure 14.

**Figure 16:**
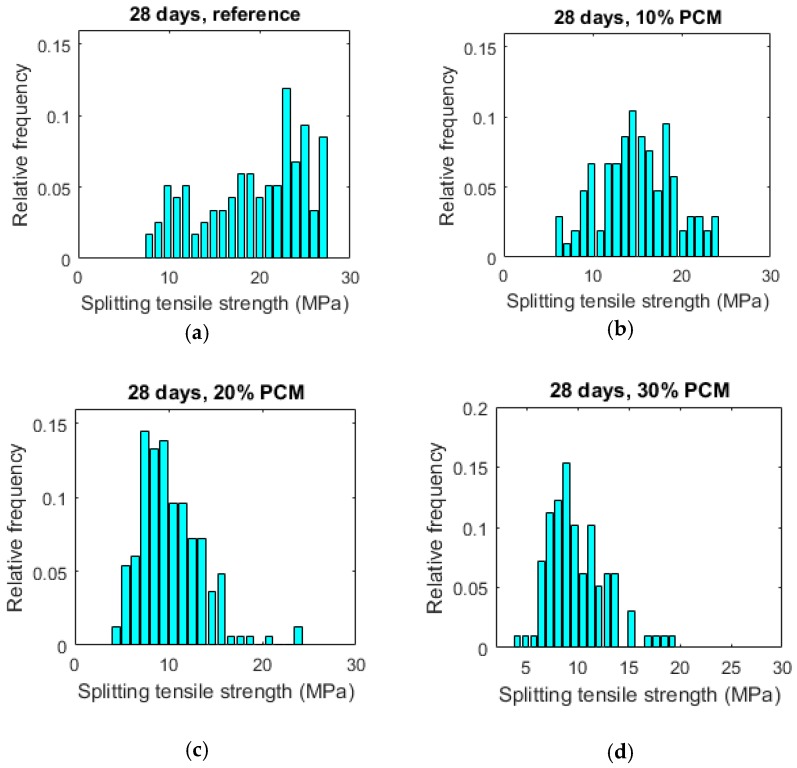
Histograms of splitting tensile strengths of micro-cubes made of pastes with various percentage of PCM microcapsules. (**a**) 28 days, reference paste; (**b**) 28 days, 10% PCM paste; (**c**) 28 days, 20% PCM paste; (**d**) 28 days, 30% PCM paste.

**Figure 17:**
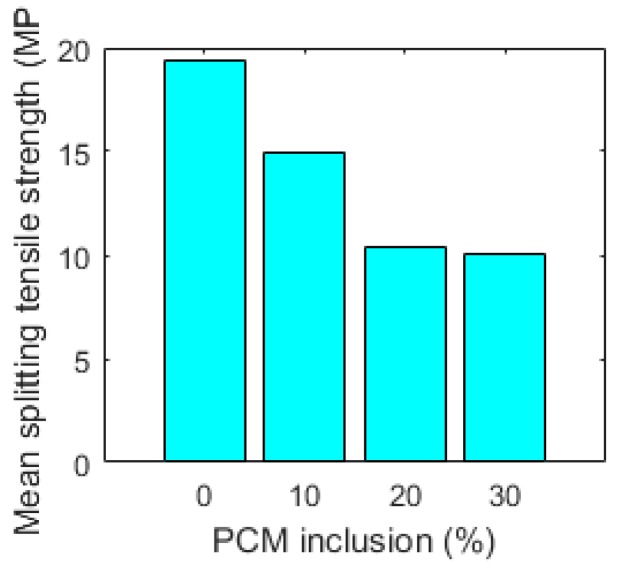
Mean splitting tensile strength of micro-cubes made of cement paste as a function of PCM volume fraction, measured by micro-cube splitting.

For each mixture, a large number of micro-cubes were fabricated and tested as previously described. The results are summarized in Table 1. Histograms of splitting tensile strengths of measured micro-cubes are given in Figure 16. From Table 1 it can be seen that mean splitting tensile strength of cement micro-cubes decreases with the increasing PCM inclusion percentage. This is shown in Figure 17.

Histograms of splitting tensile strengths shift toward lower values with increasing PCM percentages. This is accompanied by a narrower distribution, signified in a lower standard deviation (Table 1). This means that paste specimens with lower PCM contents are stronger on average but also have more weak spots (represented by weak micro-cubes) compared to the specimens with higher PCM inclusion percentages. This probably explains a difference between micro-scale results obtained in this work and tests on larger (mortar) specimens employing similar materials: for example, Ref. [13] found that flexural strength of mortar specimens incorporating (similar) PCM microcapsules is only marginally affected by PCM addition, while the compressive strength is markedly lower. Unlike the elastic modulus of composite materials which is influenced by the properties of material components and their relative amounts, the (fracture) strength is also governed by the weakest link in the system. It is therefore desirable to analyse the obtained results using Weibull statistics. The probability of failure can then be written as [40]:

(5) $$P_{f}=1-\exp[-(\frac{\sigma }{\sigma_{0}}{)}^{m}]$$

**Figure 18:**
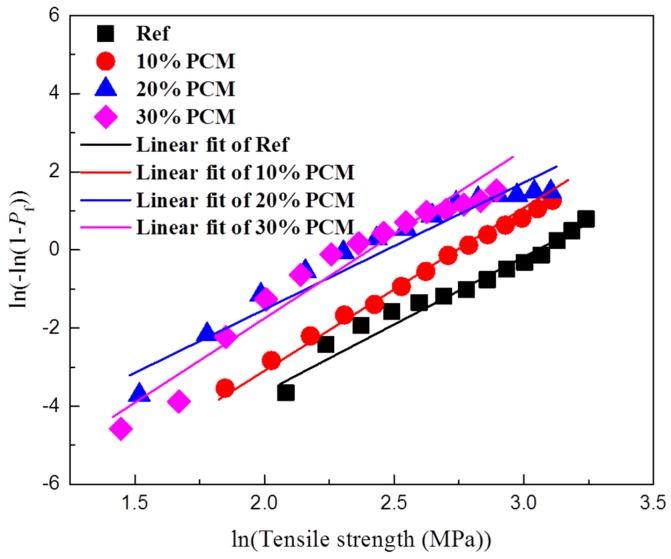
Weibull plot for measured splitting tensile strength of cement paste micro-cubes with different PCM inclusion percentages.

Here, *P_f_* is the probability of failure, *m* the Weibull modulus, and *σ*_0_ the scaling parameter (i.e., the stress corresponding to 63% probability of failure). Figure 18 shows the micro-cube splitting tensile strength tests for the tested cement pastes in a Weibull coordinate system.

All tested mixtures show a good linear fit, with a coefficient of determination (R^2^) higher than 0.95. The Weibull modulus and the scaling parameter for the tested pastes were fitted using the least squares method and are given in Table 2. The Weibull modulus increases with the increase in PCM inclusion percentage, with the exception of the 20% PCM specimen which does not follow this trend. This signifies a decrease in variability in measured micro-cube strength values for pastes with increasing inclusion percentages. This is also evident in a lower standard deviation, as given in Table 1. The scaling parameter obtained from the analysis (Table 2) shows a decrease with the increase in PCM inclusion percentage, similar to the previously shown trend for the mean splitting strength (Table 1). This analysis indicates that, although on average there is a large decrease of micro-cube splitting tensile strengths with increasing PCM inclusion percentage, the macroscopic tensile strength is not that different because it is governed by the weakest link in the system [41]. Furthermore, previous studies [8] have shown that the addition of compliant PCM microcapsules increases the toughness of the matrix by crack deflection and the microcapsule deformation ability. Since it was also shown recently that the inclusion of PCM microcapsules does not negatively affect the volume stability of cement-based composites [36], it is unlikely that it will increase shrinkage induced cracking either.

## 4. Summary and Conclusions

In this work, a detailed micromechanical characterization of cement pastes incorporating microencapsulated phase change materials (PCMs) has been performed. It was shown that the microcapsules used were spherical with a relatively fine particle size, enabling good dispersion in the cementitious matrix during the mixing process. Compression testing of individual microcapsules showed a linear relationship between the rupture force and the capsule diameter. Furthermore, it showed that there is a temperature dependence of the rupture force: capsules tested below the phase change temperature (when the core is solid) needed a higher force to rupture compared to capsules tested above the phase change temperature (when the core is liquid). 

Then, cement pastes with varying PCM inclusion percentages (0–30% per volume) were prepared and characterized. As expected, compressive strength of cement pastes showed a reduction with increasing PCM inclusion percentages for pastes aged up to 28 days. Porosity of cement pastes was characterized by MIP, showing an increase in total percolated porosity with increasing PCM addition level. This increase was much more pronounced for early ages (3 and 7 days), and relatively minor for 28 day old paste specimens. Therefore, it was concluded that the change in porosity is probably only a minor factor causing decrease in strength with increasing PCM inclusion percentages. Furthermore, the critical pore diameter, which is an important parameter governing transport properties and durability of cement based materials, was shown to be independent of the PCM inclusion percentage but dependent on hydration age. This is consistent with recent studies showing that PCM microcapsule addition does not have a detrimental effect on durability of cementitious composites [36]. Nanoindentation of 28 day old cement pastes has shown a decrease in elastic modulus with increasing PCM percentages, consistent with previous studies [8]. This was attributed mainly to the addition of compliant inclusions in the form of PCM microcapsules. Furthermore, a new micro-cube splitting technique was used to characterize splitting strength of cement pastes with varying percentages of PCM inclusions on the micro-metre length scale, which is an appropriate length scale for testing the complex micromechanical properties of concrete’s binding phase. It was found that, although pastes with higher PCM inclusion percentages showed a significantly lower average micro-cube splitting strength, the scatter in the measurements (i.e., standard deviation) was also lower. Consequently, pastes with lower PCM percentages have a relatively higher percentage of weak spots (in this case a percentage of micro-cubes weaker than the average), leading to their lower macroscopic tensile strength. This is considered to be a reason that the macroscopic tensile or flexural strength was found to be much less affected by the PCM addition compared to the compressive strength [8,9]. It should be noted, however, that due to the size of micro-cubes (100 × 100 × 100 µm), the size of microcapsules contained in these specimens was limited.

This study focused on small-scale characterization of cement pastes with PCM inclusions. Cementitious composites with PCM inclusions can be used as smart materials in a variety of applications: to promote thermal comfort in building applications [42], to melt ice and snow [11], or mitigate early and late age cracking [10,31]. Each of these applications can be achieved by adjusting the phase change temperature, the amount of phase change microcapsules and their latent heat. This study provides a basis for future developments of cementitious composites incorporating phase change materials for a variety of applications.

## Figures and Tables

**Table 1:** Summary of micro-cube splitting results.

Mixture	Number of Micro-Cubes Tested	Mean Splitting Strength (MPa)	Standard Deviation (MPa)
Reference	118	19.39	5.68
10% PCM	105	14.98	4.19
20% PCM	166	10.35	3.41
30% PCM	98	10.03	2.92

**Table 2:** Weibull parameters for the measured micro-cube splitting tensile strength.

Mixture	Number of Micro-Cubes Tested	Weibull Modulus, m	Scaling Parameter, σ_0_ (MPa)
Reference	118	3.26	21.16
10% PCM	105	4.17	15.50
20% PCM	166	3.24	11.85
30% PCM	98	4.31	11.12

## References

[1] Van Mier J.G. (2012). Concrete Fracture: A Multiscale Approach.

[2] Šavija B. (2014). Experimental and Numerical Investigation of Chloride Ingress in Cracked Concrete. Ph.D. Thesis.

[3] De Schutter G. (1999). Quantification of the influence of cracks in concrete structures on carbonation and chloride penetration. Mag. Concr. Res..

[4] Blagojević A. (2016). The Influence of Cracks on the Durability and Service Life of Reinforced Concrete Structures in Relation to Chloride-Induced Corrosion. Ph.D. Thesis.

[5] Qian S., Zhou J., De Rooij M., Schlangen E., Ye G., Van Breugel K. (2009). Self-healing behavior of strain hardening cementitious composites incorporating local waste materials. Cem. Concr. Compos..

[6] Šavija B., Luković M., Schlangen E. (2016). Influence of Cracking on Moisture Uptake in Strain-Hardening Cementitious Composites. J. Nanomech. Micromech..

[7] Bentz D.P., Turpin R. (2007). Potential applications of phase change materials in concrete technology. Cem. Concr. Compos..

[8] Fernandes F., Manari S., Aguayo M., Santos K., Oey T., Wei Z., Falzone G., Neithalath N., Sant G. (2014). On the feasibility of using phase change materials (PCMs) to mitigate thermal cracking in cementitious materials. Cem. Concr. Compos..

[9] Šavija B., Luković M., Kotteman G.M., Figuieredo S.C., de Mendoça Filho F.F., Schlangen E. (2017). Development of ductile cementitious composites incorporating microencapsulated phase change materials. Int. J. Adv. Eng. Sci. Appl. Math..

[10] Šavija B., Schlangen E. (2016). Use of phase change materials (PCMs) to mitigate early age thermal cracking in concrete: Theoretical considerations. Constr. Build. Mater..

[11] Farnam Y., Krafcik M., Liston L., Washington T., Erk K., Tao B., Weiss J. (2015). Evaluating the use of phase change materials in concrete pavement to melt ice and snow. J. Mater. Civ. Eng..

[12] Aguayo M., Das S., Castro C., Kabay N., Sant G., Neithalath N. (2017). Porous inclusions as hosts for phase change materials in cementitious composites: Characterization, thermal performance, and analytical models. Constr. Build. Mater..

[13] Aguayo M., Das S., Maroli A., Kabay N., Mertens J.C., Rajan S.D., Sant G., Chawla N., Neithalath N. (2016). The influence of microencapsulated phase change material (PCM) characteristics on the microstructure and strength of cementitious composites: Experiments and finite element simulations. Cem. Concr. Compos..

[14] Hunger M., Entrop A., Mandilaras I., Brouwers H., Founti M. (2009). The behavior of self-compacting concrete containing micro-encapsulated phase change materials. Cem. Concr. Compos..

[15] Zhang H., Šavija B., Chaves Figueiredo S., Lukovic M., Schlangen E. (2016). Microscale Testing and Modelling of Cement Paste as Basis for Multi-Scale Modelling. Materials.

[16] Koopman M., Gouadec G., Carlisle K., Chawla K., Gladysz G. (2004). Compression testing of hollow microspheres (microballoons) to obtain mechanical properties. Scr. Mater..

[17] Scrivener K., Snellings R., Lothenbach B. (2015). A Practical Guide to Microstructural Analysis of Cementitious Materials.

[18] Diamond S. (2000). Mercury porosimetry: An inappropriate method for the measurement of pore size distributions in cement-based materials. Cem. Concr. Res..

[19] Zhang J., Scherer G.W. (2011). Comparison of methods for arresting hydration of cement. Cem. Concr. Res..

[20] Oliver W.C., Pharr G.M. (2004). Measurement of hardness and elastic modulus by instrumented indentation: Advances in understanding and refinements to methodology. J. Mater. Res..

[21] Constantinides G., Ulm F.-J., Van Vliet K. (2003). On the use of nanoindentation for cementitious materials. Mater. Struct..

[22] Luković M., Šavija B., Dong H., Schlangen E., Ye G. (2014). Micromechanical study of the interface properties in concrete repair systems. J. Adv. Concr. Technol..

[23] Šavija B., Luković M., Hosseini S.A.S., Pacheco J., Schlangen E. (2015). Corrosion induced cover cracking studied by X-ray computed tomography, nanoindentation, and energy dispersive X-ray spectrometry (EDS). Mater. Struct..

[24] Ulm F.-J., Vandamme M., Jennings H.M., Vanzo J., Bentivegna M., Krakowiak K.J., Constantinides G., Bobko C.P., Van Vliet K.J. (2010). Does microstructure matter for statistical nanoindentation techniques?. Cem. Concr. Compos..

[25] Zheng K., Lukovic M., De Schutter G., Ye G., Taerwe L. (2016). Elastic Modulus of the Alkali-Silica Reaction Rim in a Simplified Calcium-Alkali-Silicate System Determined by Nano-Indentation. Materials.

[26] Luković M., Schlangen E., Ye G. (2015). Combined experimental and numerical study of fracture behaviour of cement paste at the microlevel. Cem. Concr. Res..

[27] Chen S.J., Duan W.H., Li Z.J., Sui T.B. (2015). New approach for characterisation of mechanical properties of cement paste at micrometre scale. Mater. Des..

[28] Němeček J., Králík V., Šmilauer V., Polívka L., Jäger A. (2016). Tensile strength of hydrated cement paste phases assessed by micro-bending tests and nanoindentation. Cem. Concr. Compos..

[29] Liu D., Flewitt P.E. (2017). Deformation and fracture of carbonaceous materials using in situ micro-mechanical testing. Carbon.

[30] Šavija B., Liu D., Smith G., Hallam K.R., Schlangen E., Flewitt P.E. (2016). Experimentally informed multi-scale modelling of mechanical properties of quasi-brittle nuclear graphite. Eng. Fract. Mech..

[31] Arora A., Sant G., Neithalath N. (2017). Numerical simulations to quantify the influence of phase change materials (PCMs) on the early-and later-age thermal response of concrete pavements. Cem. Concr. Compos..

[32] Young B.A., Falzone G., Zhenye S., Thiele A., Wei Z., Neithalath N., Sant G., Pilon L. (2017). Early-age temperature evolutions in concrete pavements containing microencapsulated phase change materials. Constr. Build. Mater..

[33] Lv L., Schlangen E., Yang Z., Xing F. (2016). Micromechanical Properties of a New Polymeric Microcapsule for Self-Healing Cementitious Materials. Materials.

[34] Yu Z. (2015). Microstructure Development and Transport Properties of Portland Cement-fly Ash Binary Systems: In View of Service Life Predictions. Ph.D. Thesis.

[35] Halamickova P., Detwiler R.J., Bentz D.P., Garboczi E.J. (1995). Water permeability and chloride ion diffusion in Portland cement mortars: Relationship to sand content and critical pore diameter. Cem. Concr. Res..

[36] Wei Z., Falzone G., Wang B., Thiele A., Puerta-Falla G., Pilon L., Neithalath N., Sant G. (2017). The durability of cementitious composites containing microencapsulated phase change materials. Cem. Concr. Compos..

[37] Liu D., Šavija B., Smith G.E., Flewitt P.E.J., Lowe T., Schlangen E. (2017). Towards understanding the influence of porosity on mechanical and fracture behaviour of quasi-brittle materials: Experiments and modelling. Int. J. Fract..

[38] Rocco C., Guinea G.V., Planas J., Elices M. (1999). Size effect and boundary conditions in the Brazilian test: Experimental verification. Mater. Struct..

[39] Zhang H., Šavija B., Schlangen E. (2017). Micro-cube splitting test for tensile strength characterisation of cement paste at micro scale. Constr. Build. Mater..

[40] Pizette P., Martin C., Delette G., Sornay P., Sans F. (2010). Compaction of aggregated ceramic powders: From contact laws to fracture and yield surfaces. Powder Technol..

[41] Bažant Z.P., Planas J. (1997). Fracture and Size Effect in Concrete and Other Quasibrittle Materials.

[42] Snoeck D., Priem B., Dubruel P., De Belie N. (2016). Encapsulated Phase-Change Materials as additives in cementitious materials to promote thermal comfort in concrete constructions. Mater. Struct..

